# Complete Genome Sequence of the Environmental Burkholderia pseudomallei Sequence Type 131 Isolate MSHR1435, Associated with a Chronic Melioidosis Infection

**DOI:** 10.1128/genomeA.00072-18

**Published:** 2018-03-15

**Authors:** Jason W. Sahl, Mark Mayo, Erin P. Price, Derek S. Sarovich, Mirjam Kaestli, Talima Pearson, Charles H. D. Williamson, Roxanne Nottingham, Krystal Sheridan, David M. Wagner, Bart J. Currie, Paul Keim

**Affiliations:** a Pathogen and Microbiome Institute, Northern Arizona University, Flagstaff, Arizona, USA; b Menzies School of Health Research, Darwin, Australia

## Abstract

The Burkholderia pseudomallei isolate MSHR1435 is a fully virulent environmental sequence type 131 (ST131) isolate that is epidemiologically associated with a 17.5-year chronic melioidosis infection. The completed genome will serve as a reference for studies of environmental ecology, virulence, and chronic B. pseudomallei infections.

## GENOME ANNOUNCEMENT

Burkholderia pseudomallei is the causative agent of melioidosis, a usually acute disease, with ∼50% of patients presenting with pneumonia and with an overall mortality rate of 10 to 40% (1). Chronic melioidosis (defined as symptoms being present for more than 2 months before diagnosis) accounts for around 10% of presentations (2). The longest continuous chronic melioidosis infection to be reported is patient 314 (P314) from the Darwin Prospective Melioidosis Study (2), who was initially diagnosed in June 2000 with a sputum culture-positive infection and who remains sputum culture positive as of August 2017 (3). Analysis of 3 clinical P314 genomes (MSHR1655, MSHR1043, and MSHR6686) deposited in GenBank (4) demonstrated substantial mutations and genome deletions with attenuated virulence, explaining the persisting chronic carriage without clinical deterioration (3). Here, we describe the completed genome sequence of MSHR1435, isolated in 2002 from water samples near the home of P314 in the Northern Territory, Australia. The close genetic similarity between the P314 clinical genomes and MSHR1435 suggests that this strain reflects B. pseudomallei environmental strains that were involved in the initial infection in P314. Clinical strains show mutations in genes associated with virulence, suggesting that MSHR1435 represents a model strain to better understand the genomic mechanisms behind B. pseudomallei pathogenesis in this unique-to-date chronic infection scenario.

Total genomic DNA was extracted using the DNeasy blood and tissue kit from Qiagen, with an attempt to limit mechanical shearing. For PacBio sequencing, approximately 10 µg of DNA was fragmented to 10 to 20 kbp using the g-TUBE apparatus (Covaris). The sequencing library was constructed using the SMRTbell template prep kit 1.0 and according to the PacBio 20-kb library protocol. Sequencing was performed on the PacBio RS II instrument in one single-molecule real-time (SMRT) cell (version 3) for 6 h. Illumina libraries were constructed with 300-bp inserts using standard methods. The genome was assembled with Canu version 1.3 (5), with 6 iterations of Pilon version 1.22 (6) to correct small indels, resulting in 2 contigs representing the 2 chromosomes in B. pseudomallei. The genome was reoriented with Circlator version 1.4.0 (7) and annotated with the NCBI Prokaryotic Genome Annotation Pipeline (PGAP) version 4.3 (8).

The chromosomes of MSHR1435 were 4,019,555 (chromosome 1) and 3,258,775 (chromosome 2) nucleotides (nt) in size, with an average GC content of 67.9%. PGAP predicted 6,946 coding genes, 4 complete *rrn* operons, and 60 tRNAs. A screen of virulence genes previously identified in B. pseudomallei using the large-scale BLAST score ratio (LS-BSR) (9) pipeline demonstrated the presence of the Burkholderia thailandensis-like flagellum and chemotaxis (BTFC) gene cluster (10), lipopolysaccharide (LPS) genotype A (11), an intact *wcbR* gene associated with capsular polysaccharide synthesis (12), the filamentous hemagglutinin gene, *fhaB3*, associated with positive blood cultures (13), and intact type III (14) and type VI secretion systems (15). A study of stepwise mutations in clinical strains from P314 compared to MSHR1435 will provide insight into the genomic mechanisms associated with attenuated virulence and chronic colonization in B. pseudomallei.

### Accession number(s).

This whole-genome assembly has been deposited at DDBJ/ENA/GenBank under accession numbers CP025264 and CP025265. Raw data were deposited in the Sequence Read Archive (SRA) for both PacBio (SRR6413814) and Illumina (SRR5314868) read data.
